# Cost-of-Illness in Psoriasis: Comparing Inpatient and Outpatient Therapy 

**DOI:** 10.1371/journal.pone.0078152

**Published:** 2013-10-23

**Authors:** Sabine I. B. Steinke, Wiebke K. Peitsch, Alexander Ludwig, Matthias Goebeler

**Affiliations:** 1 Department of Dermatology, University Hospital Münster, University of Münster, Münster, Germany; 2 Department of Dermatology, University Medical Centre Mannheim, University of Heidelberg, Heidelberg, Germany; 3 Center for Macroeconomic Research, University of Cologne, Cologne, Germany; 4 Munich Research Institute for the Economics of Aging, Max Planck Institute Munich, Munich, Germany; 5 Department of Dermatology, Venereology and Allergology, University Hospital Würzburg, University of Würzburg, Würzburg, Germany; Ludwig-Maximilian-University, Germany

## Abstract

Treatment modalities of chronic plaque psoriasis have dramatically changed over the past ten years with a still continuing shift from inpatient to outpatient treatment. This development is mainly caused by outpatient availability of highly efficient and relatively well-tolerated systemic treatments, in particular BioLogicals. In addition, inpatient treatment is time- and cost-intense, conflicting with the actual burst of health expenses and with patient preferences. Nevertheless, inpatient treatment with dithranol and UV light still is a major mainstay of psoriasis treatment in Germany. The current study aims at comparing the total costs of inpatient treatment and outpatient follow-up to mere outpatient therapy with different modalities (topical treatment, phototherapy, classic systemic therapy or BioLogicals) over a period of 12 months. To this end, a retrospective cost-of-illness study was conducted on 120 patients treated at the University Medical Centre Mannheim between 2005 and 2006. Inpatient therapy caused significantly higher direct medical, indirect and total annual costs than outpatient treatment (13,042 € versus 2,984 €). Its strong influence on cost levels was confirmed by regression analysis, with total costs rising by 104.3% in case of inpatient treatment. Patients receiving BioLogicals produced the overall highest costs, whereas outpatient treatment with classic systemic antipsoriatic medications was less cost-intense than other alternatives.

## Introduction

Psoriasis is a chronic inflammatory disease affecting about 2% of the population in Europe and Northern America. In Germany, an estimated 1.6 million individuals suffer from this disease that implicates considerable physical, psychological and social burden for patients but also a major financial burden for health economy [1,2]. Annual costs for psoriasis treatment come up to 7 billion € in Germany and up to 11 billion US-$ in the United States [3–6]. In the US, moderate to severe psoriasis is mostly treated in an outpatient setting [7,8], while in Germany and other European countries inpatient treatment, mostly conducted as a regimen of topical dithranol combined with UV light, still is a common, but expensive therapeutic alternative [9–12]. However, this regimen is increasingly being replaced by highly effective outpatient alternatives, in particular by biologicals [4,13–16]. 

Given the rising expenses for health, economic analyses comparing the different treatment modalities are imperative. Such analyses performed in the Angloamerican context mostly concentrated on cost efficiency of different outpatient alternatives [5,17–19], as inpatient therapy does not play a major role in these regions. During the last years some European studies have examined costs for psoriasis treatment including inpatient therapy in detail [14,20–26]. However, they often do not consider inpatient therapy as a separate treatment alternative to be compared with outpatient therapy or they only include selected outpatient treatments. Comprehensive cost analyses considering the available treatment options for outpatients in comparison to inpatients are lacking so far. We therefore initiated this study to compare inpatient therapy with all available outpatient treatments from an economic point of view. 

## Materials and Methods

### Data collection

The present cost-of-illness analysis was carried out as a 12-month retrospective bottom-up study with a separate view of inpatient and outpatient therapy. Three hundred ninety-one patients treated for psoriasis between January 2005 and July 2006 either as inpatients or outpatients at the Department of Dermatology of the University Medical Centre Mannheim, University of Heidelberg, were contacted by mail in December 2006. Of 202 patients answering back (return rate: 51.7%), 149 gave written informed consent to participate. One hundred twenty patients were available for personal interviews and could be included in the study. The personal interview was conducted by one interviewer from February until July 2007 using a 17-page standardized questionnaire (see Questionnaire S1), focussing on sociodemographic characteristics (age, sex, annual income, employment status, weekly working hours, insurance), treatments received for psoriasis (in- or outpatient therapy, treatment mode, duration, frequency and location, number of clinic or office visits, diagnostic procedures, kind and number of prescriptions, package sizes), individual treatment costs (co-payments for prescriptions, costs for therapies not covered by the insurance including skin care products and private treatments at health resorts, transportation expenses) as well as days absent from work due to psoriasis. Moreover, detailed information on duration and frequency of in- and outpatient treatment and visits, prescribed treatments, diagnostic procedures and sick certificates was collected from the medical records, together with medical information such as Psoriasis Area and Severity Index (PASI), Dermatology Life Quality Index (DLQI), presence or absence of psoriatic arthritis and co-morbidities. Disease severity was classified using the maximal PASI recorded in the investigation period in conjunction with the classification criteria of the Committee for Medicinal Products for Human Use (CHMP; 1). For individuals treated merely as outpatients, costs and medical details were assessed for a 12-month period between January 2005 and July 2006. For inpatients, corresponding data were collected for the months following hospital demission during outpatient care. 

### Ethics statement

The study was conducted according to the ethics principles expressed in the Declaration of Helsinki and approved by the Medical Ethics Committee II of the University Medical Centre Mannheim, University of Heidelberg, Mannheim, Germany. All patients gave written informed consent for participation in the study.

### Sample stratification

For statistical analysis the cohort (n=120 patients) was stratified into inpatients and outpatients as well as according to the most intense outpatient treatment modality, i.e. topical therapy, phototherapy (PUVA, narrowband UVB 311 nm, selective UV therapy), classic systemic therapy (methotrexate, fumaric acid, cyclosporine, retinoids) or biological therapy (infliximab, etanercept or efalizumab). The majority of patients (68%) received a combination of two or more treatment modalities (for details, see Table 1). For example, in the group “classic systemic therapy” 9 of 12 outpatients were prescribed topical treatment in addition to a classic systemic agent and one patient was treated with a triple combination of classic systemic medication, topical therapy and phototherapy. Seventy-one individuals were treated merely as outpatients, with 28 receiving topical therapy, 28 phototherapy, 12 classic systemic therapy and 3 biologicals as most intense therapy. Forty-nine patients were admitted as inpatients at least once during the observation period. In the year following inpatient treatment eight patients received only topical therapy, 21 phototherapy, 15 classic systemic therapy and 5 biologicals as most intense treatment (Table 1).

**Table 1:** Sample stratification according to the most intense outpatient treatment.

**Grouping by maximal outpatient therapy**	**Inpatients (n=49)**	**Outpatients (n=71)**
**Topical therapy**	**8 (16.3%)**	**28 (39.4%)**
**Phototherapy**	**21 (42.9%)**	**28 (39.4%)**
Phototherapy only	1	0
Phototherapy + topical therapy	20	28
**Classic systemic therapy**	**15 (30.6%)**	**12 (16.9%)**
Classic systemic therapy only	0	2
Classic systemic therapy + topical therapy	9	9
Classic systemic therapy + phototherapy + topical therapy	6	1
**Biological therapy**	**5 (10.2%)**	**3 (4.2%)**
Biological therapy only	0	0
Biological therapy + topical therapy	3	2
Biological therapy + classic systemic therapy	1	0
Biological therapy + classic systemic therapy + topical therapy	1	0
Biological therapy + classic systemic therapy + phototherapy + topical therapy	0	1

### Cost definition and calculation

Costs were classified into direct medical, direct non-medical and indirect costs, according to economic analyses from the societal perspective [27]. Direct medical costs were costs for inpatient treatment, outpatient medication, consultation and diagnostics as well as for skin care products. These costs were assessed according to DRG (“Diagnosis Related Groups”) and EBM (”Einheitlicher Bewertungsmaßstab”, Uniform Value Scale) guidelines valuable in 2006 as well as to the valid medication prices in Germany (as referenced in the “Red List”). Direct non-medical costs included costs for travel expenses or for private treatments at health resorts. These costs were directly generated from the personal interview. Indirect costs, accruing from loss of working time, were estimated from the mean gross income of the individual subject according to the human capital approach. The human capital approach is one of the most frequently used methods for estimating indirect costs through loss of working time. As one major advantage, e.g. in contrast to the friction cost approach, the human capital approach assigns losses of reduced honorary activities caused by illness an equal monetary value.

### Statistical analysis

Data were analyzed using SPSS 12.0 and Microsoft Office Excel 2003 with *t*-tests, variance analysis (ANOVA, Welch, Brown-Forsythe) and multivariate regression analysis. In the latter, logarithmic values for direct medical costs, costs for outpatient medication, costs for outpatient visits and diagnostics, indirect costs through loss of working time and total costs were taken as dependent variables. After logarithmic transformation Kolmogoroff-Smirnoff tests presented normal distribution of all cost variables (Table S1). Logarithmic calculus is a well-known approach for variables which cannot take values <0. Besides, it allows comprehensive interpretation of regression results, as interpretation of point estimates as changes in percent or percentage points is only possible with logarithmic dependent variables. 

Independent variables were age, sex, income, employment status, PASI, DLQI and disease duration. Dummy variables for inpatient therapy (reference group: outpatient therapy), for phototherapy and systemic therapy (reference group: topical therapy) as well as for full time employment, part time employment and minor employment (reference group: not working) were incorporated. All independent variables were concomitantly integrated into the analysis.

## Results

### Demographic and clinical characteristics

Among the patients included into the study, 55% were male and 45% female (Table 2). The mean age was 51.8 years. Forty percent of the patients worked full time (>37.5 hours per week) and 13.3% part time (19.5-37.5 hours per week) whereas 40% had no employment at all because of retirement (25.8%), unemployment (9,2%) or homemaking (8,3%). The vast majority had compulsory health insurance.

**Table 2:** Demographic and clinical characteristics of patients included in the study.

		**Mean [range]**		
		**All (n=120)**	**Inpatients (n=49)**	**Outpatients (n=71)**
Age [years]		51.8 [19-82]	51.5 [25-82]	52.0 [19-79]
Gender		m 55%, f 45%	m 61%, f 39%	m 51%, f 49%
Employment status	Full time employment	40% (n=48)	42.9% (n=21)	38% (n=27)
	Part time employment	13.3% (n=16)	8.2% (n=4)	16.9% (n=12)
	Minor employment	6.7% (n=8)	6.1% (n=3)	7% (n=5)
	Unemployment	9.2% (n=11)	14.3% (n=7)	5.6% (n=4)
	Retirement	25.8% (n=31)	22.4% (n=11)	28.2% (n=20)
	Homemaking	8.3% (n=10)	10.2% (n=5)	7.0% (n=5)
Health insurance	Compulsory	94.2% (n=113)	93.9% (n=46)	94.4% (n=67)
	Other	5.8% (n=7)	6.1% (n=3)	5.6% (n=4)
Disease duration [years]		22.0 [1-72]	24.8 [1-72]	20.1 [1-57]
Type of psoriasis	Psoriasis vulgaris	90%	89.8%	90.1%
	Psoriasis guttata	3.3%	4.1%	0%
	Psoriasis palmoplantaris	6.7%	12.3%	2.8%
PASI Score		10.8 [0-67.2]	13.5 [0-67.2]	8.9 [0.6-34.5]
DLQI Score		9.2 [0-27]	10.2 [0-27]	8.4 [0-26]
Disease severity according to CHMP criteria	Light - moderate	29.1%	22.4%	33.8%
	Moderate - severe	26.7%	32.7%	22.5%
	severe	44.2%	44.8%	43.7%
Psoriatic arthritis	Established diagnosis	15.8%	20.4%	12.7%
	History of arthralgias	19.2%	14.3%	22.5%
Other comorbidities	None	31.7% (n=38)	22,4% (n=11)	38% (n=27)
	1	19.2% (n=23)	22.4% (n=11)	16.9% (n=12)
	2	28.3% (n=34)	26.5% (n=13)	29.6% (n=21)
	>2	20.8% (n=25)	28.7% (n=14)	15.5% (n=11)
Days absent from work due to psoriasis		14.8	32.7	2.4
Outpatient consultations		10.8	12.7	9.5
Time for skin care per week [hours]		4.1	4.9	3.5
Loss of leisure time per week [hours]		6.6	8.8	5.0

Ninety percent of the patients suffered from plaque psoriasis, and the mean disease duration was 22 years. Mean PASI scores were 13.5 for inpatients and 8.9 for mere outpatients. According to the classification criteria of the Committee for Medicinal Products for Human Use [1], more than 70% suffered from moderate or severe psoriasis (Table 2). 

When inpatients were compared to outpatients, the former had a longer disease duration with an earlier onset, more severe psoriasis (as assessed by PASI, p=0.002, *t*-test) and a higher impact on life quality as assessed by DLQI (*t*-test, not significant).

Inpatients were absent from work for more than one month during the assessment period whereas mere outpatients only lost two working days due to psoriasis (Table 2; p<0.001, *t*-test). After demission from hospital former inpatients sought medical consultation for psoriasis somewhat more frequently than mere outpatients (12.7 versus 9.5 consultations per year, *t*-test, not significant). 

In case of inpatient treatment, the mean length of a single hospital stay at our department was 19.6 days. A considerable number of patients (26.5%) required more than one hospital stay during the 12-month observation period leading to a mean total hospitalization time of 27.9 days. Eighty-four percent of inpatients were treated with dithranol and phototherapy. Mean patient-reported remission time after inpatient treatment was 104.3 days. Remarkably, despite intense topical and phototherapy during the hospital stay a high percentage of inpatients required phototherapy (55%), classic systemic therapy (35%) or biologicals (10%) after demission.

Regarding the different treatment modalities prescribed in an outpatient setting, either for mere outpatients or as follow-up therapy after inpatient treatment, 93% of all patients were treated with topical therapy, including medical skin care products, 45% with phototherapy, 25% with classic systemic therapy and 6.7% with biologicals. Details of the different regimens are indicated in Table 3. In 68% of all patients a combination of different treatment modalities was recommended, most frequently a combination of phototherapy or classic systemic therapy with topical therapy (Table 1). 

**Table 3:** Treatment regimens of the patient collective (n=120).

	**n**	**[%] of n=120**		**n**	**[%]**
**Topical therapy**	112	93,3	Vitamin D	88	78,6
			Salicylic acid	66	58,9
			Corticosteroids	62	55,4
			Vitamin D and corticosteroids	57	50,9
			Urea	29	25,9
			Anthralin	28	25,0
			Magistral formulas	18	16,1
			Retinoids	10	8,9
**Phototherapy**	54	45	Creme- resp. Shower-PUVA	28	51,9
			UVB 311nm	24	44,4
			Light comb	17	31,5
			UVA/UVB	12	22,2
**Classic systemic therapy**	30	25	Retinoids	10	33,3
			Methotrexate	10	33,3
			Fumaric acid	8	26,7
			Cyclosporine	3	10,0
**Biological therapy**	8	6,7	Etanercept	3	37,5
			Infliximab	3	37,5
			Efalizumab	2	25,0

### Cost analysis of the whole collective

When costs were analyzed for the whole patient collective, mean total annual costs per patient added up to 7,092 €, including average direct medical costs of 4,978 €, direct non-medical costs of five hundred and ninety-eight € and indirect costs through loss of working time of 1,515 € (Table 4). In the category of direct medical costs inpatient treatment accounted for the highest expenses (2,311 € per patient and year, 46.4% of all direct medical costs), followed by expenses for outpatient medication (1,987 €, 39.9%). In contrast, costs for outpatient visits and diagnostics as well as costs for skin care were comparatively low.

**Table 4:** Comparison of annual costs for inpatient and outpatient therapy.

	**Costs per patient per year [€]**		
	Mean		
	Median [P25, P75]		
	**All (n=120)**	**Inpatients (n=49)**	**Outpatients (n=71)**
**Direct medical costs**	**4,978**	**9,511**	**1,851**
	**2,664 [1,296, 6,164]**	**6,793 [5,366, 11,061]**	**1,496 [733, 1,988]**
Inpatient therapy	2,311	5,660	-
	0 [0, 4,170]	4,171 [4,114, 8,098]	-
Outpatient medication	1,987	3,078	1,234
	850 [446, 1471]	942 [588, 2,666]	757 [335, 1331]
Costs for outpatient visits and diagnostics	210	241	189
	133 [85, 266]	152 [84, 310]	128 [84, 249]
Costs for skin care	470	531	427
	360 [180, 600]	360 [240, 660]	324 [120, 600]
**Direct non-medical costs**	**598**	**559**	**625**
	**101 [24, 579]**	**96 [26, 551]**	**108 [24, 654]**
**Indirect costs**	**1,515**	**2,973**	**509**
	**0 [0, 2,188]**	**2,210 [1,217, 3,956]**	**0 [0, 0**]
**Total costs**	**7,092**	**13,042**	**2,985**
	**4,122 [1,508, 10,244]**	**10,286 [7,153, 16,244]**	**1,721 [1,038, 3,435]**

### Cost comparison between inpatients and outpatients

For further cost analysis and comparison the cohort was stratified into inpatients and outpatients. Total annual costs for inpatients were more than as fourfold high as those for mere outpatients (Table 4; 13,042 € versus 2,985 €; p<0.001, *t*-test). This was due to (a) higher total direct medical costs including costs for hospitalization (9,511 € vs. 1,851 €; p<0.001, *t*-test) and (b) higher indirect costs through loss of working time (2,973 € vs. five hundred and nine €; p<0.001, *t*-test). Unexpectedly, however, costs for outpatient medication in the time following hospitalization were also significantly higher for former inpatients than for mere outpatients (3,078 € vs. 1,234 €; p=0.026, *t*-test), leading to more than twofold higher direct medical costs for ambulatory treatment (3,850 € versus 1,850 €; p=0.021, *t*-test).

### Cost comparison according to different treatment modalities

Stratification according to the different treatment modalities (topical therapy, phototherapy, classic systemic therapy, biologicals, Tables 5 and 6) revealed that patients receiving biologicals as most intense treatment produced several fold higher total annual costs than patients treated with all other modalities, both in the inpatient (30,200 €; Table 5) and in the outpatient group (11,601 €; Table 6). This was mainly attributable to high medication costs. In addition, therapy with biologicals appeared to be associated with considerably higher indirect costs due to loss of working time (Tables 5 and 6). The latter may at least partially be explained by the application of infliximab in 3 of 8 patients that needs to be delivered as infusion in an office- or clinic-based setting. However, because of the small number of patients receiving biologicals (n=5 in the inpatient and n=3 in the outpatient cohort), statistical significances could not be calculated for this subgroup.

**Table 5:** Cost comparison for inpatients stratified according to the maximal follow-up outpatient therapy.

	**Costs per patient per year [€]**			
	Mean			
	Median [P25, P75]			
	**Inpatient therapy (n=49)**			
	**Topical (n=8)**	**Photo (n=21)**	**Systemic (n=15)**	**Biological (n=5)**
**Direct medical costs**	**6,189**	**7,320**	**9,491**	**24,083**
	**5,150 [4,262, 7,896]**	**5,725 [5,259, 9,641]**	**7,939 [5,616, 10,518]**	**23,661 [20,420, 27,958]**
Inpatient therapy	5,430	5,411	6,366	4,954
	4171 [3,387, 7305]	4,171 [4,114, 8,163]	4,171 [4,114, 8,098]	4,171 [3,666, 6,633]
Outpatient medication	406	1,206	2,233	17,756
	454 [22, 630]	942 [540, 1,835]	1,316 [757, 3,633]	18,026 [12,952, 22,432]
Costs for outpatient visits and diagnostics	91	122	358	625
	93 [29, 151]	115 [79, 174]	332 [220, 492]	446 [178, 1,154]
Costs for skin care	263	581	533	749
	240 [120, 405]	420 [279, 720]	360 [260, 780]	600 [282, 1,290]
**Direct non-medical costs**	**181**	**340**	**853**	**1,196**
	**64 [18, 330]**	**58 [21, 318]**	**498 [75, 864]**	**444 [38, 2,729]**
**Indirect costs**	**2,141**	**2,231**	**3,807**	**4,921**
	**1,955 [796, 3,309]**	**1,877 [549, 3,525]**	**2,605 [1,658, 4,148]**	**3,956 [3,213, 7,112]**
**Total costs**	**8,512**	**9,891**	**14,151**	**30,200**
	**6,944 [5,389, 12,478]**	**8,168 [6,781, 13,615]**	**10,504 [8,491, 17,511]**	**26,400 [24,501, 37,798]**

**Table 6:** Cost comparison for mere outpatients stratified according to their maximal therapy.

	**Costs per patient per year [€]**			
	Mean			
	Median [P25, P75]			
	**Outpatient therapy (n=71)**			
	**Topical (n=28)**	**Photo (n=28)**	**Systemic (n=12)**	**Biological (n=3)**
**Direct medical costs**	**1,197**	**1,509**	**2,223**	**9,652**
	**889 [309, 1,576]**	**1,519 [1,015, 1,933]**	**1,942 [1,448, 3,128]**	**10,785 [6,691, .**]
Inpatient therapy	-	-	-	-
Outpatient medication	674	860	1,557	8,718
	385 [129, 950]	750 [529, 1,209]	1,329 [822, 2,254]	9,853 [5,679, .]
Costs for outpatient visits and diagnostics	127	153	317	551
	103 [57, 128]	134 [85, 214]	256 [218, 410]	532 [259, .]
Costs for skin care	396	496	349	383
	270 [120, 585]	342 [120, 738]	330 [180, 525]	480 [70, .]
**Direct non-medical costs**	**1,121**	**347**	**198**	**297**
	**189 [40, 986]**	**69 [20, 324]**	**136 [33, 260]**	**274 [244, .**]
**Indirect costs**	**116**	**958**	**95**	**1,651**
	**0 [0, 0**]	**0 [0, 0**]	**0 [0, 0**]	**182 [0, .**]
**Total costs**	**2,434**	**2,813**	**2,517**	**11,601**
	**1,199 [449, 3,293]**	**1,655 [1,141, 2,234]**	**2,160 [1,513, 3,446]**	**11,706 [11,160, .**]

Both in the inpatient and in the outpatient group, classic systemic therapy produced significantly higher direct medical costs in the outpatient sector than topical or phototherapy (3,124 € resp. 2,223 €, p<0.001, *t*-tests; Tables 5 and 6). This was attributable both to significantly higher expenses for outpatient visits and diagnostics (three hundred and fifty-eight € resp. three hundred and seventeen €, p<0.001, *t*-tests; Tables 5 and 6) and to significantly higher costs for outpatient medication (2,233 € resp. 1,557 €, p=0.006 for the inpatient group, p=0.015 for the mere outpatient group, *t*-tests). 

Direct non-medical costs, including e.g. costs for private treatments at health resorts or travel expenses, were somewhat higher for outpatients on topical therapy than for those on phototherapy or classic systemic therapy (1,121 € vs. three hundred and forty-seven € vs. one hundred and ninety-eight €; Table 6). However, these differences were not statistically significant (p=0.174 resp. p=0.102, *t*-tests). Furthermore, outpatients receiving phototherapy had higher indirect costs due to loss of working time than those receiving topical or systemic therapy (p=0.297 resp. p=0.485, *t*-tests, not significant; Table 6). 

Overall, the highest cost categories were costs for inpatient treatment, for outpatient medication and indirect costs for loss of working time. Remarkably, costs that have to be covered by the individual, i.e. co-payments for medication, costs for skin care products and treatments not covered by the insurance as well as travel expenses were considerable, ranging from four hundred and forty-four € to 1,944 € per patient per year. 

### Regression analysis

To analyse the influence of treatment modalities on different cost categories, we performed a regression analysis (Table 7). Costs for inpatient therapy were compared to those for mere outpatient therapy and costs for classic systemic and phototherapy to those for topical therapy. Due to the small sample size, patients receiving biologicals had to be excluded from these analyses (n=112 for regression analysis). Cost variables were normally distributed according to one-sample Kolmogoroff-Smirnoff test when using logarithmic values (Table S1). Direct medical costs were predicted to rise by 127.8% (p<0.001) and total costs by 104.3% (p<0.001) if choosing the inpatient therapy option instead of mere outpatient therapy. By contrast, costs for outpatient medication, visits and diagnostics were predicted to decrease by 10.0% or by 31.6%, respectively, in case of inpatient treatment.

**Table 7:** Regression analysis showing high impact of treatment mode on cost levels (n=112; n=8 patients treated with biologicals excluded because of group size).

	**Increase of costs [%**]		
	**Inpatient therapy**	**Phototherapy**	**Classic systemic therapy**
	**(n=44)**	**(n=49)**	**(n=27)**
***Reference group***	***Outpatienttherapy***	***Topicaltherapy***	
	***(n=68)***	***(n=36)***	
**Direct medical costs**	127,8***	41,3**	60,3***
**Outpatient medication costs**	-10,0	78,4**	103,9***
**Costs for outpatient visits and diagnostics**	-31,6	30,8*	108,3***
**Indirect costs through loss of working time**	2,7	43,8**	7,3
**Total costs**	104,3***	30,7*	44,1*

^*^ p<0.05

^**^ p<0.01

^***^ p<0.001

Comparing the influence on cost levels if phototherapy or classic systemic therapy were chosen instead of mere topical therapy, all direct medical costs were predicted to rise significantly (Table 7). The greatest increase was noted for costs for outpatient medication (103.9%, p<0.001) and costs for outpatient visits and diagnostics (108.3%, p<0.001) in case of classic systemic therapy. Indirect costs increased significantly only in case of phototherapy, as expected from our descriptive cost analysis. Also in accordance to the descriptive data, total annual costs raised when phototherapy (30.7%, p=0.030) or classic systemic therapy (44.1%, p=0.011) were used instead of mere topical treatment. 

## Discussion

In the light of rising costs for health care, economic analyses of treatment strategies are becoming increasingly important. Several studies have been performed to compare the cost-effectiveness of selected treatment alternatives for psoriasis, recently focussing on biologicals [28–30]. Furthermore, trends in direct medical costs for psoriasis have been analyzed in detail, comparing UV therapy, classic systemic agents and biologicals and showing a cost increase at a considerable higher rate than general inflation for almost all agents and modalities [31]. However, all-encompassing cost analyses also including indirect costs are rare. Moreover, only few studies comprise inpatients, as this alternative is rather uncommon in many Western countries. We here provide a comprehensive cost analysis of all important costs categories and all available treatment alternatives in form of a bottom-up study with a separate view on inpatient and outpatient therapy.

### Comparison with other cost analyses

A few respective analyses also including inpatients have been performed in Germany [21,20,22,23; Table S2]. However, none of them comprised patients treated with biologicals, as data were collected before the broad introduction of these agents. The mean total annual costs determined in the German studies by Sohn et al. [23] and Schöffski et al. [22] were slightly lower compared to our study (7,092 € in our study versus 6,709 or 6,707 €, respectively; Table S2). This is mainly attributable to slightly higher costs for inpatient treatment and somewhat higher indirect costs noted in our study. 40.8% of our patients, but only 28.3% of the patients analyzed in the two other studies were hospitalized in the observation period, but the average total hospitalization time was considerably shorter in our collective (27.9 versus 39.1 days). Considering that our collective comprised patients on biologicals and the other collective did not, it is remarkable that costs for outpatient medication were similar. However, the number of patients on biologicals included in our study was low (n=8) and these therapies were only prescribed for an average of four months during the assessment period.

In another German multicenter study by Berger and colleagues [20] total annual costs were remarkably lower (2,866 €; Table S2), mostly due to lower direct medical costs. This may be explained by the study population investigated and by the study design since (a) only a very low percentage of the patients (2.6%) were hospitalized and (b) data were collected only for 3 months retrospectively and 6 weeks prospectively and subsequently extrapolated to the year-horizon. 

Total costs, total direct medical costs and indirect costs due to loss of working time were also assessed in a large US American study by Fowler and colleagues, comprising 12,280 patients treated for psoriasis between 1998 and 2005 [32]. Cost levels were similar to those of our study, with the exception of somewhat higher indirect costs, including “disability payments” to be provided by the employer. However, none of these reports explicitly compared in- and outpatient therapy as separate options; neither does an Italian study form the pre-biological era that comprises large numbers of inpatients [25]. 

### Costs of biological therapy

In contrast to phototherapy and classic systemic therapy, costs for biologicals clearly exceeded costs for inpatient treatment. This effect was appreciable from the descriptive data, despite the small number of patients on biologicals that prohibited statistical analysis for this subgroup. Our analysis is likely to underestimate total actual costs of biological therapy, as in the index years 2005 and 2006 most study patients just started to take biologicals, which had been introduced in Germany only shortly before (i.e. in autumn 2004). Patients received these therapies for an average of only four months within the assessment period. Today, there is a trend to long-term therapy with biologicals with presumably higher direct medical costs. Moreover, medication costs for most biologicals have been rising considerably more than the general inflation rate over the last years [31].

Also according to other recent studies, total costs increased after introduction of biologicals, as the rise in medication costs could not fully outweigh possible decreases in costs for hospitalizations or outpatient admissions [21,26,31,34].

In a German study comparing inpatient treatment to outpatient biological therapy with efalizumab, total annual costs for the patient group receiving efalizumab were more than threefold higher than for the inpatient group [21]. However, this study might have underestimated follow-up costs after inpatient treatment. Furthermore, efalizumab, now withdrawn from the market, is known to be by far less effective than other biologicals.

Fonia et al. [26] showed an increase of total annual health care costs by £7,774 per patient after introduction of biologic agents. Mean annual inpatient and outpatient admission costs were hereby reduced by £1,682 whilst mean annual drug costs increased by £9,456. Indirect costs were not examined.

In a study by Ghatnekar et al. [34] biologicals were used in 16% of the patients and accounted for 20% of the total costs, whereas traditional systemic drugs (methothrexate, acitretin and cyclosporine) were prescribed for 26% of the patients and accounted for only 0.5% of total costs. 

Only in the American study of Bhosle et al. [35], which was conducted shortly after the introduction of biologicals total health care costs did not differ significantly from the pre-biologics period (14,662.22 US-$ versus 16,156.10 US-$; p>0.05) despite a significant rise in prescription costs (33). 

Overall, introduction of biologicals into the treatment of psoriasis has led to a considerable raise in costs. However, patients are significantly more satisfied and compliant with biologicals than with other treatments [37]. Moreover, the higher costs for biologicals also might outweigh long-term health care costs of psoriasis patients, as biologicals have favourable effects on the long term course of psoriatic arthritis and probably also beneficial effects on concomitant cardiovascular diseases.

### Role of inpatient treatment

Our study demonstrates impressively that costs for inpatient therapy exceed costs for all conventional outpatient alternatives by far. Remarkably, former inpatients also required high expenses for outpatient follow-up treatments, with 55% needing additional phototherapy, 35% classic systemic therapy and 10% biologicals. According to our data inpatient therapy may still be less expensive than biological therapy. However, inpatient treatment with dithranol and UV light cannot substitute for biological treatment, as the effect of inpatient treatment only had a considerably short duration of less than four months. As a consequence, several inpatients of our cohort were prescribed biologicals after demission. Our primary hypothesis that time- and cost-intense inpatient treatment might lead to long term remission of psoriasis and therefore to decreased follow-up costs had to be rejected.

The enormous costs produced by inpatient therapy may in part be justified by the fact that inpatients were more severely affected than mere outpatients, with higher PASI scores and higher prevalence of psoriatic arthritis and comorbidities (Table 2). It was recently shown that comorbidities are associated with significantly higher hospitalization rates, outpatient visits and total costs [33]. 

Willingness of patients to be hospitalized is decreasing as effective and well-tolerated outpatient treatment options are becoming accessible. In a recent study analysing patient preferences for psoriasis treatments it was shown that patients attach great value to outpatient treatment [36]. The trend towards long-term outpatient treatment will continue, and further highly efficient biological treatments such as, e.g., interleukin-17 antagonists are expected to become available in the near future. Nevertheless, inpatient treatment will probably retain its role for management of psoriasis patients with multiple comorbidities and/or with acute exacerbation. 

### Strengths and limitations

Our study has several limitations. As data were collected shortly after the introduction of biologicals into the German market, the subgroup of patients receiving biologicals was too small to explicitly compare costs for outpatient biological therapy to inpatient therapy with appropriate statistical methods. Our cost data stem from 2005/2006. Since then the price of etanercept slightly decreased by 2-3%, whereas the price for infliximab increased by 8-19%. In addition, some of the most efficient biologicals, i.e. adalimumab and ustekinumab, were not available at the time of data collection. 

Our study was conducted as a monocentric cost analysis, impairing generalization of our results. Moreover, direct comparison of inpatient and outpatient costs is hampered by the fact that inpatients were more severely affected than outpatients and presented higher PASI scores, higher prevalence of psoriatic arthritis and higher rates of comorbidities. 

The well-known human capital approach was used for measuring indirect costs, as it allowed the inclusion of the societal value of honorary activities. With regard to the high proportion of unemployment within our study collective (40%) that approach seemed to estimate indirect costs in the most appropriate way. Nevertheless this approach tends to overestimate opportunity costs in the long run, why a supplement by e.g. a friction cost approach could have been reasonable.

A major strength of our study is that it provides the first all-encompassing cost analysis including all available treatment modalities. Our analysis reflects the real-world cost situation, in which prescription of combination regimens is frequent. Costs have been collected not only by chart review, allowing detailed calculation of direct medical costs, but also in personal interviews, which provided precise information on individual costs and indirect costs. 

## Conclusion

Our study shows that the costs of inpatient therapy are significantly higher than those of outpatient treatment. Inpatient treatment did not result in decreased follow-up costs, as the duration of remission was relatively short and several patients required systemic treatment after demission. Patients receiving biologicals produced the overall highest costs. Topical treatment, phototherapy or classic systemic medications were significantly less costly when prescribed in an outpatient setting, as expected. Mere outpatient treatment with classic systemic anti-psoriatic medications turned out to be less cost-intense than phototherapy. Despite high requirements for laboratory testing and monitoring for adverse effects, this option is attractive from the economic perspective.

## Supporting Information

Questionnaire S1 Cost-of-illness in Psoriasis (original German version).
(PDF)

Table S1 Costs are normally distributed when using logarithmic values (n=112; n=8 patients treated with BioLogicals excluded because of group size).
(DOC)

Table S2 Comparison to other German cost-of-illness studies.
(DOC)
