# Correction: Cost-of-Illness in Psoriasis: Comparing Inpatient and Outpatient Therapy 

**DOI:** 10.1371/annotation/cf71846b-1fa5-46bb-ac82-d5884b17451d

**Published:** 2014-01-17

**Authors:** Sabine Steinke, Wiebke K. Peitsch, Alexander Ludwig, Matthias Goebeler

The name of the first author was given incorrectly. The correct name is: Sabine Steinke. The correct citation is: Steinke S, Peitsch WK, Ludwig A, Goebeler M (2013) Cost-of-Illness in Psoriasis: Comparing Inpatient and Outpatient Therapy. PLoS ONE 8(10): e78152. doi:10.1371/journal.pone.0078152. 

The correct abbreviation in the Author Contributions Statement is: SS.

