# Supplementary material for: Cost-of-Illness in Psoriasis: Comparing Inpatient and Outpatient Therapy
Source: PLoS One. 2013 Oct 23;8(10):e78152. doi: 10.1371/journal.pone.0078152 (PMC3806808; doi:10.1371/journal.pone.0078152)
Supplement: Questionnaire S1 — Cost-of-illness in Psoriasis (original German version). (PDF) [file pone.0078152.s001.pdf]

KLINIKUM MANNHEIM
Universitätsklinikum gGmbH
Medizinische Fakultät Mannheim der Universität Heidelberg

KLINIK FÜR DERMATOLOGIE
VENEROLOGIE UND ALLERGOLOGIE
DIREKTOR:
PROF. DR. S. GOERDT

Ambulante und stationäre Therapie der Psoriasis – ein ökonomischer Kostenvergleich aus gesellschaftlicher Perspektive

Wir freuen uns sehr, dass Sie sich die Zeit nehmen, um an unserer Studie zur Psoriasis teilzunehmen. Die Psoriasis ist eine Volkskrankheit, an der in Deutschland über 1 Million Menschen leiden. Die Lebensqualität des Einzelnen kann durch die Psoriasis erheblich eingeschränkt sein. Zudem entstehen durch die Krankheit nicht nur hohe gesellschaftliche Kosten, sondern auch erhebliche finanzielle und nicht-finanzielle Aufwendungen für die Betroffenen selbst.

Im Rahmen dieser Studie wollen wir uns mit Auswirkungen der Psoriasis sowohl auf die Lebensqualität als auch auf die Kosten, die für die einzelnen Patienten und die Gesellschaft durch die Erkrankung entstehen, beschäftigen.

Beginnen werde ich mit einigen Fragen zu Ihrer Person.

A_Demografische Daten

**1.**	**Wann sind Sie geboren? (TT/MM/JJJJ)**<br>______________________________
**2.**	**Welches Geschlecht haben Sie?**<br>Weiblich………………………………❑<br>Männlich………………………………❑
**3.**	**Welche Nationalität haben Sie?**<br>Deutsch………………………………❑<br>Italienisch……………………………❑<br>Türkisch………………………………❑<br>Sonstige: ________________________
**4.**	**In welcher familiären Situation befinden Sie sich momentan?**<br>Verheiratet,<br>zusammen lebend………………❑<br>Verheiratet,<br>dauernd getrennt lebend…………❑<br>Verwitwet………………………………❑<br>Geschieden……………………………❑<br>Alleinstehend…………………………❑

5. **Wie viele Personen (incl. Ihnen selbst) leben in Ihrem Haushalt?**

____

Wieviele Kinder haben Sie?

____

Wieviele Ihrer Kinder befinden sich momentan in Ausbildung?

____

6. **Welcher ist Ihr höchster Schulabschluss?**

- Abitur……………………………………☐
- Fachhochschulreife……………………☐
- Mittlere Reife, Realschulabschluss…☐
- Hauptschulabschluss…………………☐
- Sonstiger Abschluss……………………☐
- Kein Abschluss…………………………☐

7. **Welchen Beruf haben Sie erlernt?**

- Gelernter Beruf: ______________________________
- Keinen…………………………………☐

Welchen Ausbildungsweg haben Sie hierfür gewählt?

- Lehre/ Ausbildung……………………☐
- Studium (Uni/FH/BA)…………………☐
- Sonstige…………………………………☐

8. **In welcher beruflichen Stellung sind Sie zurzeit beschäftigt?**

- Beruf: ______________________________
- Arbeiter…………………………………☐
- Angestellter……………………………☐
- Selbständig……………………………☐
- Auszubildender…………………………☐
- Rentner…………………………………☐
- Mutterschutz……………………………☐
- Hausfrau/ Hausmann…………………☐
- Wehrdienst/Zivildienst/FSJ……………☐
- Arbeitslos………………………………☐
- Sonstiges: ______________________________

**9.**	**Wieviele Stunden arbeiten Sie durchschnittlich pro Woche in Ihrem Hauptberuf?**<br>Vertragliche Arbeitszeit: ______________________________<br>Tatsächliche Arbeitszeit: ______________________________
**10.**	**Üben Sie derzeit eine Erwerbstätigkeit aus? Was trifft für Sie zu?**<br>Voll erwerbstätig……………………….❑<br>In Teilzeitbeschäftigung…..…………….❑<br>In betrieblicher Ausbildung/ Lehre<br>oder Umschulung………………….❑<br>Geringfügig oder unregelmäßig<br>erwerbstätig……………………….❑<br>Nicht erwerbstätig…………………….❑

11. Wie hoch ist Ihr monatliches Nettoeinkommen in Euro?

Individual-EK: ____________________

< 500………………………………… ☐

500 – 1000……………………………… ☐

> 1000 – 1500………………………… ☐

> 1500 – 2000 ………………………… ☐

> 2000 – 2500 ………………………… ☐

> 2500 – 3000 ………………………… ☐

> 3000 – 3500 ………………………… ☐

> 3500 – 4000 ………………………… ☐

> 4000 – 5000 ………………………… ☐

> 5000 – 7500 ………………………… ☐

> 7500 – 10000 ……………………… ☐

> 10000 ……………………………… ☐

Haushalts-EK: ____________________

< 500………………………………… ☐

500 – 1000……………………………… ☐

> 1000 – 1500………………………… ☐

> 1500 – 2000 ………………………… ☐

> 2000 – 2500 ………………………… ☐

> 2500 – 3000 ………………………… ☐

> 3000 – 3500 ………………………… ☐

> 3500 – 4000 ………………………… ☐

> 4000 – 5000 ………………………… ☐

> 5000 – 7500 ………………………… ☐

> 7500 – 10000 ……………………… ☐

> 10000 ……………………………… ☐

12. Wie hoch ist Ihr monatliches Brutto-Einkommen in €?

Individual-EK: ____________________

< 500…………………………………… ☐

500 – 1000……………………………… ☐

> 1000 – 1500………………………… ☐

> 1500 – 2000 ………………………… ☐

> 2000 – 2500 ………………………… ☐

> 2500 – 3000 ………………………… ☐

> 3000 – 3500 ………………………… ☐

> 3500 – 4000 ………………………… ☐

> 4000 – 5000 ………………………… ☐

> 5000 – 7500 ………………………… ☐

> 7500 – 10000 ……………………… ☐

> 10000 ………………………………… ☐

Haushalts-EK: ____________________

< 500…………………………………… ☐

500 – 1000……………………………… ☐

> 1000 – 1500………………………… ☐

> 1500 – 2000 ………………………… ☐

> 2000 – 2500 ………………………… ☐

> 2500 – 3000 ………………………… ☐

> 3000 – 3500 ………………………… ☐

> 3500 – 4000 ………………………… ☐

> 4000 – 5000 ………………………… ☐

> 5000 – 7500 ………………………… ☐

> 7500 – 10000 ……………………… ☐

> 10000 ………………………………… ☐

**13.**	**Sind Sie gesetzlich oder privat krankenversichert?**<br>Gesetzlich……………………………… ☐<br>Privat…………………………………… ☐<br>Bei welcher Kasse?<br>____<br>Wie hoch ist Ihr monatlicher Beitrag (Arbeitnehmeranteil) in €?<br>____<br>Sind Sie …<br>Beitragszahlendes Pflichtmitglied…… ☐<br>Beitragszahlendes Familienmitglied… ☐<br>Mitversichertes Familienmitglied……. ☐<br>Versichert als Rentner/ Arbeitsloser/<br>Student/ Wehr- oder<br>Zivildienstleistender…………… ☐<br><br>**Haben Sie eine private Zusatzversicherung abgeschlossen?**<br>Ja……………………………………… ☐<br>Nein…………………………………… ☐<br>Wenn ja, welche? __________________________________<br>Und wofür?_______________________________________<br>Wie hoch ist der monatliche Beitrag in €?<br>____
**14.**	**Rauchen Sie gegenwärtig?**<br>Ja………………………………………… ☐<br>Nein………………………………………. ☐<br>Falls Nein, haben Sie früher geraucht?<br>Ja………………………………………… ☐<br>Nein………………………………………. ☐

Es existieren ja viele verschiedene Formen und Schweregrade der Psoriasis. Im Folgenden werde ich Ihnen nun zu Ihrer Erkrankung ein paar Fragen stellen.

B_Medizinische Daten

15. An welcher Form der Psoriasis sind Sie erkrankt?

- Plaque-Psoriasis (Psoriasis vulgaris)……..☐
- Psoriasis guttata……………………………☐
- Psoriasiserythrodermie……………………..☐
- Psoriasis capitis……………………………☐
- Psoriasis pustulosa generalista……………☐
- Psoriasis palmoplantaris……………………☐
- sonstige……………………………………..☐

16. Besteht bei Ihnen eine Psoriasis-Arthritis?

- Ja……………………………………………..☐
- Nein…………………………………………..☐
- Gesichert……………………………………☐
- Vermutet……………………………………..☐

17. Wie hoch ist Ihr aktueller PASI?

____

18. Wie hoch ist Ihr maximaler PASI im Beobachtungszeitraum (2006 bei rein ambulanter Behandlung bzw. im laufenden Jahr nach der stationären Behandlung)?

____

19. Wie hoch ist Ihr aktueller DLQ-Index?

____

20. Wann sind bei Ihnen zum ersten Mal Symptome einer Psoriasis aufgetreten?

Alter:______________________________________

Jahr: ______________________________________

Wann ist bei Ihnen zum ersten Mal die Diagnose „Psoriasis“ gestellt worden?

Alter:______________________________________

Jahr: ______________________________________

**21.**	**Wie viele Tage pro Jahr haben Sie durchschnittlich Symptome der Psoriasis?**<br>______________________________<br><br>**Wie viele Tage hatten Sie im letzten Jahr Symptome der Psoriasis (2006 bei rein ambulanter Behandlung bzw. im laufenden Jahr nach stationärer Behandlung)?**<br>______________________________
**22.**	**Welche Erkrankungen haben Sie zusätzlich zur Psoriasis?**<br>Arterielle Hypertonie…………………………☐<br>Fettstoffwechselstörung……………………..☐<br>Koronare Herzerkrankung/ Angina pectoris..☐<br>Herzinfarkt……………………………………☐<br>Andere Herzerkrankung……………………..☐<br>Darmerkrankung (z.B. M. Crohn)…………….☐<br>Diabetes mellitus……………………………..☐<br>Lebererkrankung………………………………☐.<br>Streptokokkeninfekte (z.B. Angina)…………☐<br>Sonstige: ________________________<br>______________________________<br>Keine:…………………………………………☐

Die Therapie der Psoriasis kann sowohl stationär als auch ambulant durchgeführt werden. Hierbei können sich die Kosten und der Aufwand für den einzelnen Patienten erheblich unterscheiden. Im Folgenden werde ich Ihnen mehrere Fragen zu Ihrer Psoriasistherapie stellen.

C_Stationäre Behandlung

23. Wurden Sie 2005 oder 2006 stationär wegen der Psoriasis behandelt?

Ja……………………………………………☐
Nein…………………………………………☐ → weiter mit Frage 27

Wie lange war Ihr stationärer Aufenthalt insgesamt in Tagen?

____

24. Welche Therapie hatten Sie während des stationären Aufenthaltes?

____

25. Hat sich Ihre Psoriasis-Erkrankung nach dem stationären Aufenthalt verändert?

Ja……………………………………………☐
Nein…………………………………………☐

Falls ja, wie hat sich Ihre Psoriasis-Erkrankung durch den stationären Aufenthalt verändert?

Sehr verbessert…………………………☐
Verbessert………………………………☐
Etwas verbessert………………………☐
Gleichgeblieben…………………………☐
Etwas verschlechtert……………………☐
Verschlechtert……………………………☐
Sehr verschlechtert……………………☐

Falls eine Verbesserung eingetreten ist, wie lange hielt diese nach Beendigung des stationären Aufenthaltes an (in Wochen)?

____

Hat sich Ihre Lebensqualität nach dem stationären Aufenthalt verändert?

Ja………………………………………… ☐

Nein…………………………………….… ☐

Falls ja, wie hat sich Ihre Lebensqualität durch den stationären Aufenthalt verändert?

Sehr verbessert……………………….… ☐

Verbessert……………………………… ☐

Etwas verbessert…………………….… ☐

Gleichgeblieben………………………. ☐

Etwas verschlechtert………………… ☐

Verschlechtert…………………………. ☐

Sehr verschlechtert…………………… ☐

26. DRG-Nummern

____

D_Ambulante Behandlung

27. **Wurden Sie 2005 oder 2006 ambulant wegen der Psoriasis behandelt?**

Ja………………………………………☐

Nein……………………………………☐

28. **Wie oft waren Sie 2006 bei rein ambulanter Behandlung bzw. im laufenden Jahr nach stationärer Behandlung bei einem Arzt zur Behandlung der Psoriasis?**

1 x………………………………………☐

2 x………………………………………☐

3 x………………………………………☐

4 x………………………………………☐

5 x………………………………………☐

6 x………………………………………☐

7 x………………………………………☐

8 x………………………………………☐

9 x………………………………………☐

10 x……………………………………☐

11 x……………………………………☐

12 x……………………………………☐

>12 x…………………………………☐

29. **Welche und wie viele unterschiedliche Ärzte haben Sie wegen der Psoriasiserkrankung aufgesucht (Arztpraxen oder Kliniken/Ambulanzen)?**

____

____

30. Diagnostik

Labor:

Differential-Blutbild internistischer Block: ______________

RF, CCP:____

ASL, AST, ASK, ADNase: _________________________

ANA, dsDNS: ____________________________________

Hepatitis A, B, C: _________________________________

Röntgen:

Gelenke: _______________________________________

Hände: ____

Füße: ____

Knie: ____

Hände in Weichstrahltechnik: _______________________

Thorax: ____

Nebennasenhöhle, Zähne: _________________________

2-Phasen-Knochen-Szintigraphie: ___________________

Tb-Test: ____

Oberbauch-Sonographie: ___________________________

C 13-Harnstoff-Atemtest: ____________________________

Sonstige:

____

Konsile

Rheumatologie: ____________________________________

HNO: ____

31. Mit welchen verschiedenen Lokaltherapien wurden Sie im Beobachtungszeitraum (2006 bei rein ambulanter Behandlung oder im laufenden Jahr nach stationärer Behandlung) behandelt?

	Medikament und Dosis	Dauer (Tage)	Anzahl Verschreibungen	Größe
1.	________	____	____	____
2.	________	____	____	____
3.	________	____	____	____
4.	________	____	____	____
5.	________	____	____	____
6.	________	____	____	____
7.	________	____	____	____
8.	________	____	____	____

32. Wurden Sie mit Lichttherapie behandelt? Wenn ja, mit welcher?

Ja……………………………………☐
Nein………………………………….☐
UVB 311 nm………………………..☐
Dusch-PUVA………………………..☐
Creme- oder Pinsel- PUVA…………☐
Lichtkamm…………………………….☐
SOP…………………………………….☐
Sonstige……………………………….☐

Mit welcher Frequenz? ___________________________________

Wie lange dauerte die einzelne Behandlung in Minuten?

____

Wo erhielten Sie die Behandlung?

Praxis………………………….. ❑

Klinik stationär…………………. ❑

Klinik ambulant ………………… ❑

33. **Wurden Sie mit systemischer Therapie behandelt? Wenn ja, mit welchem Medikament und welcher maximalen Dosis? Wie lange dauerte die Behandlung und wie oft wurde Ihnen das Medikament in welcher Größe verschrieben?**

Ja…………………………………………….❑

Nein…………………………………………❑

	Medikament und max. Dosis	Dauer (Tage)	Anzahl Verschreibungen	Größe
1.	______________________	_______	_________	__________
2.	______________________	_______	_________	__________
3.	______________________	_______	_________	__________
4.	______________________	_______	_________	__________

Erhielten Sie eine Begleitmedikation?

Ad 1:	____________________	_______	_________	___________
Ad 2:	____________________	_______	_________	___________
Ad 3:	____________________	_______	_________	___________
Ad 4:	____________________	_______	_________	___________

Hatten Sie Nebenwirkungen? Wie wurden diese therapiert?<br>Ad 1: ________________________ ________________<br>Ad 2: ________________________ ________________<br>Ad 3: ________________________ ________________<br>Ad 4: ________________________ ________________

Psoriasis-Patienten entstehen oft zusätzliche Kosten wie z.B. für Pflegeprodukte. Auch können Sie sich in Ihrem alltäglichen Leben eingeschränkt fühlen. Auf diese Aspekte beziehen sich nun die letzten Fragen.

E_Indirekte Kosten

**34.**	**Wie hoch waren Ihre persönlichen monatlichen Ausgaben 2006 bzw. im Jahr nach der stationären Behandlung für… (in €)**<br>Hautpflege:________________________________<br>Spezielle Kleidung:___________________________<br>Spezielle Nahrung:___________________________<br>Sonstiges:_________________________________
**35.**	**Falls Sie 2006 bei rein ambulanter Behandlung bzw. im laufenden Jahr nach stationärer Behandlung erwerbstätig waren: Wieviele Tage haben Sie wegen der Psoriasiserkrankung oder der Psoriasistherapie in Ihrer Erwerbstätigkeit nicht gearbeitet?**<br>Tage, an denen Sie gar nicht gearbeitet haben:<br>___________________________<br>Tage, an denen Sie nur teilweise gearbeitet haben:<br>___________________________
**36.**	**Wie viele Stunden benötigen Sie für häusliche Pflege Ihrer Haut auf Grund der Psoriasiserkrankung pro Woche?**<br>Selbständige Pflege:________________________________<br>Pflege durch Familienangehörigen: ______________________<br>Pflege durch eine bezahlte Hilfskraft:____________________
**37.**	**Wie lange fahren Sie durchschnittlich zu Ihrem behandelnden Arzt in Minuten?**<br>____

	**Wie viele km ist Ihr behandelnder Arzt von Ihrer Wohnung entfernt?** ____
**38.**	**Wieviele Stunden an Freizeit entgehen Ihnen pro Woche auf Grund der Psoriasiserkrankung?** ____

Herzlichen Dank für das Beantworten des Fragebogens!
