# Supplementary material for: Cost-of-Illness in Psoriasis: Comparing Inpatient and Outpatient Therapy
Source: PLoS One. 2013 Oct 23;8(10):e78152. doi: 10.1371/journal.pone.0078152 (PMC3806808; doi:10.1371/journal.pone.0078152)
Supplement: Table S1 — Costs are normally distributed when using logarithmic values (n=112; n=8 patients treated with BioLogicals excluded because of group size). (DOC) [file pone.0078152.s002.doc]

|  | **Skewness Statistic/ Standard error** | | **One Sample Kolmogoroff-Smirnoff Test** | |
| --- | --- | --- | --- | --- |
|  |  | ***Logarithmic costs*** |  | ***Logarithmic costs*** |
| **Direct medical costs** | 2.770/ 0.228 | *-0.349/ 0.228* | 0.001 | *0.225* |
| **Outpatient medication costs** | 2.569/ 0.228 | *-0.926/ 0.233* | 0.000 | *0.178* |
| **Costs for outpatient visits and diagnostics** | 1.656/ 0.228 | *-0.491/ 0.229* | 0.002 | *0.352* |
| **Indirect costs through loss of working time** | 4.467/ 0.228 | *-0.505/ 0.228* | 0.000 | *0.567* |
| **Total costs** | 2.766/ 0.228 | *-0.434/ 0.228* | 0.000 | *0.378* |

Table S1. *Costs are normally distributed when using logarithmic values (n=112; n=8 patients treated with biologicals excluded because of group size)*
