# Supplementary material for: Cost-of-Illness in Psoriasis: Comparing Inpatient and Outpatient Therapy
Source: PLoS One. 2013 Oct 23;8(10):e78152. doi: 10.1371/journal.pone.0078152 (PMC3806808; doi:10.1371/journal.pone.0078152)
Supplement: Table S2 — Comparison to other German cost-of-illness studies. (DOC) [file pone.0078152.s003.doc]

Table S2. *Comparison to other German cost-of-illness studies*

|  | **Costs per patient per year [€]** | | | | |
| --- | --- | --- | --- | --- | --- |
|  | **Direct medical costs** | **Inpatient treatment** | **Outpatient medication** | **Indirect costs** | ***Total costs*** |
| Our study | 4,978 | 2,311 | 1,987 | 1.515 | ***7,092*** |
| Schöffski et al. 2007 | 4,603 | 2,298 | 2,014 | 1,310 | ***6,707*** |
| Sohn et al. 2006 | 4,517 | 2,299 | 2,014 | 1,310 | ***6,709*** |
| Berger et al. 2005 | 1,426 | 183 | 514 | 1,440 | ***2,866*** |
